# Exploration of synthesis of anthocyanins in passion fruit pericarp through combinatorial transcriptomic analysis and WGCNA

**DOI:** 10.3389/fpls.2025.1658779

**Published:** 2025-10-09

**Authors:** Hui-Ying Chen, Fan-Li Gao, Quan Guo, Sheng-Nan Bao, Xia Li, Hock Eng Khoo

**Affiliations:** ^1^ Guangxi Key Laboratory of Electrochemical and Magneto-chemical Functional MaterialsCollege of Chemistry and Bioengineering, Guilin University of Technology, Guilin, China; ^2^ School of Food & Pharmaceutical Engineering Zhaoqing University, Zhaoqing, China

**Keywords:** transcriptome analysis, passion fruit, anthocyanins, WGCNA, RT-qPCR

## Abstract

Passion fruit (*Passiflora edulis*) is extensively cultivated in most tropical and subtropical regions worldwide. Yet although its pulp, well known for nutritional profile, is commonly processed into juice and other products, the peel remains underutilized, leading to substantial biomass wastes. This study investigates the molecular mechanisms of anthocyanin accumulation in the peel through the integrative transcriptome and WGCNA analysis. Samples were collected at both mature and immature developmental stages from purple- and yellow-skinned passion fruit varieties. Transcriptome sequencing was performed on the Illumina-Hiseq platform. After stringent quality trimming and filtering, the clean reads were aligned to the reference genome. Differentially expressed genes (DEGs) were identified using DESeq2 with the thresholds of |log2Fold Change| ≥ 1 and FDR < 0.05. To elucidate their biological functions and metabolic pathways, the DEGs were annotated against the KEGG, GO and KOG databases. Subsequent WGCNA,pinpointed ten candidate hub genes potentially involved in anthocyanin biosynthesis. Finally, their expression patterns were validated via RT-qPCR, which showed strong concordance with the transcriptome data. These findings not only confirm the robustness of our analytical approach but also highlight the pivotal regulatory roles of several key genes, involving *Pe*CA, *Pe*MYC2, *Pe*MYB88, and *Pe*CHI, in the passion fruit anthocyanin biosynthesis pathway.

## 1 Introduction

Passion fruit (*Passiflora edulis*) belongs to the genus Passiflora, a perennial evergreen herbaceous vine in the family Passifloraceae. Its juice is rich in vitamins, antioxidants, essential amino acids, lutein and minerals required 57 by the human body (Chen et al., 2025). cultivated extensively in tropical and subtropical regions, it comprises two primary commercial varieties: yellow (*P. edulis f. flavicarpa*) and purple (*P. edulis f. edulis*). Both varieties possess significant pharmaceutical and nutraceutical value. Yellow passion fruit demonstrates therapeutic potential for neurological conditions, with documented sedative, anxiolytic, and neuropharmacological effects through modulation of γ-aminobutyric acid (GABA) activity (Sena et al., 2009). Similarly, purple passion fruit exhibits diverse pharmacological properties, including anti-inflammatory, analgesic, and cardiovascular protective effects (Mota et al., 2018). Despite the economic importance of passion fruit primarily for juice production, the peel—constituting approximately 50-60% of the fruit’s fresh weight—remains largely underutilized, representing a significant agricultural waste challenge.

The peel, particularly from the purple variety, is a rich reservoir of bioactive compounds, most notably anthocyanins, the pigments responsible for its characteristic coloration (Deng et al., 2010). These polyphenolic compounds exhibit diverse biological activities, including antioxidant, anti-inflammatory, antimicrobial, and cardioprotective effects (Khoo et al., 2017). Consequently, anthocyanins are highly sought-after as natural colorants and functional ingredients in the food, pharmaceutical, and cosmetic industries. The biosynthetic pathway of anthocyanins, initiated from phenylalanine through sequential enzymatic reactions, has been well-characterized in model plants (Chemat et al., 2017). However, a comprehensive understanding of the molecular machinery governing anthocyanin accumulation in passion fruit peel remains elusive. The specific regulatory genes and co-expression networks that differentiate the high-anthocyanin purple variety from the low-anthocyanin yellow variety, especially during fruit maturation, are largely uncharacterized.

The advent of high-throughput technologies, including RNA sequencing (RNA-seq), has enabled a comprehensive and systematic investigation of the functional characteristics of thousands of genes (van Dam et al., 2015). RNA-seq is employed to profile differentially expressed genes in peels of purple and yellow passion fruits across various developmental stages. Gene co-expression network analysis is a powerful approach for elucidating the functional roles and interrelationships of genes based on genome-wide expression data (Childs et al., 2011). This methodology entails the construction of a network of co-activated genes across multiple samples.Currently, weighted gene co-expression network analysis (WGCNA) represents the most frequently utilized systems biology methodology for identifying gene correlation patterns (Tahmasebi et al., 2019). This is particularly valuable for the identification of co-expressed gene modules, establishing their correlations with phenotypic traits, and pinpointing key regulatory hub genes.

In this study, we employed an integrated transcriptomic and WGCNA analyses of peels from purple and yellow passion fruit varieties at both immature and mature developmental stages. Our primary objectives were to: (1) profile the transcriptomic changes associated with anthocyanin accumulation; (2) identify key structural and regulatory genes differentially expressed during anthocyanin accumulation; (3) construct gene co-expression networks to elucidate regulatory mechanisms; and (4) pinpoint candidate hub genes that likely play a central role in regulating anthocyanin biosynthesis. Our findings provide fundamental insights into the molecular basis of anthocyanin biosynthesis in passion fruit and establish a genomic resource for developing strategies to valorize this underutilized agricultural byproduct.

## 2 Materials and methods

### 2.1 Sample preparation

Two passion fruit cultivar utilized in this study: purple passion fruit (*Passiflora edulis* ‘Tainong No. 1’), and yellow passion fruit (*Passiflora edulis* ‘Qinmi 9’), were both collected from the demonstration orchard in the Sujia Village, located in the Wanhe Zhelmu Town, Guilin City of Guangxi region. From each cultivar, a total of 100 fruits was meticulously selected from the fruit trees, divided into four groups, and then randomly sampled. The fruit samples were then classified into two maturity categories: ripe and unripe. The peels of unripe purple passion fruit were designated as TPU, and those of ripe fruits as TPR. For the yellow passion fruit, the peels of unripe fruits were labeled as TYU, and those of ripe fruit as TYR. The fruit samples were then washed with tap water and air-dried. The pectin layer was removed from the fruit peels, followed by a thorough washing of the pericarp layer and its subsequent drying on a bench. Subsequently, the peel samples were flash-frozen using liquid nitrogen, pulverized, and stored at -80 °C for further analysis. The fruit peels from the yellow variety were used for comparison.

### 2.2 Transcriptome sequencing

Transcriptome sequencing was conducted in the UW Genetics Ltd. (Shenzhen, China). The total RNA was extracted from the peel samples of the purple passion fruit (TPU and TPR) and yellow passion fruit (TYU and TYR) using an RNA extraction kit (Cheng et al., 2023). Subsequently, Total RNA concentration and purity were measured by spectrophotometry. RNA integrity was assessed using the Agilent 2100 Bioanalyzer. After passing quality control, paired-end sequencing was performed on the Illumina HiSeq platform.

### 2.3 Data processing

The library was subjected to sequencing using a second-generation sequencing platform (Illumina HiSeq 2000). An initial evaluation of the data was then conducted using the FastQC bioinformatics tool (Ward et al., 2020). Subsequently, the results were integrated and presented in a visual format using a MultiQC software (Ewels et al., 2016). Non-genomic sequences were trimmed using NGSQC Toolkit and Trimmomatic softwares with default parameters based on the evaluation results (Bolger et al., 2014). The softwares were employed for strict control of raw data, including removals of non-genomic sequences, reads with connectors (adapters). The presence of N bases in excess of this threshold has been demonstrated to affect the subsequent comparison results. Furthermore, low-quality bases (Q ≤ 20) were removed. Next, PCR duplicates generated during library construction were removed using FastUniq software to obtain clean reads for the subsequent comparisons (Xu et al., 2012). After quality control steps, the clean reads were compared to the reference genome in order to identify the source genes that were transcribed by these fragments. The clean reads were then aligned aganist the *Passiflora* reference genome using HISAT2, which yielded information regarding the genomic location, gene features, and sequence characteristics that were specific to the sequenced sample.

### 2.4 Analysis of gene expression level

The truly differentially expressed genes (DEGs) were addressed based on their biological variability due to considerable variation in gene expression among individuals. To facilitate a comprehensive analysis of the entire transcriptome, biological replicates were established. Pearson’s correlation coefficient (R) was employed to assess the correlation between biological replicates in the transcriptome sequencing data. Pearson’s correlation coefficient (R) was also utilized to quantify the degree of correlation between biological replicates (Li et al., 2015). As R approaches 1, the correlation between the replicates increases.

In the RNA-Seq, transcript abundance functioned as an indicator of gene expression levels. FPKM (fragments per kilobase of transcript per million mapped reads) was utilized to quantify transcript abundance. This approach quantifies the expression level of each gene as a fraction of the total length of the transcript in millions of mapped reads (Mizushima et al., 2020). Principal component analysis (PCA) was a statistical method that does not require supervision. PCA was used to identify patterns in the data. The principal component 1 (PC1) represented the principal component that captured most of the variance in the data matrix, while the principal component 2 (PC2) captured additional and distinct variance. The employment of PCA was instrumental in extracting the maximum amount of information from the original variables, thereby ensuring their independence (Liu et al., 2003).

### 2.5 Identification of DEGs

The DESeq2 method was employed to analyze the differential expression among the samples studied, thereby identifying the DEG sets between groups. The unstandardized data were utilized as the input for the differential analysis. Subsequent to the differential analysis, the Benjamini-Hochberg method was employed to correct the P-values for multiple testing, thereby yielding the false discovery rate (FDR). The identification of differential genes was facilitated by establishing a criteria of [log_2_ fold change] ≥ 1 and FDR < 0.05. Subsequently, hierarchical clustering analysis was performed on FPKM expression data following differential gene centralization and standardization, and a corresponding heatmap was generated (Love et al., 2014; Varet et al., 2016).

### 2.6 Gene annotation and enrichment analysis

#### 2.6.1 KEGG annotation and pathway enrichment analysis

The KEGG (Kyoto Encyclopedia of Genes and Genomes) automatic annotation server (KAAS, http://www.genome.jp/tools/kaas/) was employed as a tool to annotate the sequences using a set of Perl scripts (Kanehisa, 2002). The Bayesian network-based Bayesian belief propagation (BBS) mode was selected for the annotation of the transcriptome sequences. Subsequently, KEGG pathway enrichment analysis was performed on the annotation results of KEGG Ortholog. The number of genes that could be annotated on the KEGG Pathway was obtained by the KAAS annotation. Finally, the Python-based software was used to group, sort, summarize, and visualize the pathways based on the hypergeometric distribution principle (Klopfenstein et al., 2018).

#### 2.6.2 GO annotation of DEGs

Gene Ontology (GO) is an international classification system for gene functions that has been used to describe and categorize gene and protein functions across different species. The categorization of GO is typically divided into three distinct categories (Chen, 2017): The first category, “Molecular Functions” (MFs), encompasses the activities that genes perform within a cell. The second category, “Biological Processes” (BPs), refers to the collective functions that organisms use to survive and reproduce. The third category, “Cellular Components” (CCs), concerns the structural components of cells. The significance of enrichment in GO terms was determined by comparing DEGs with the entire genome background using the hypergeometric test. This approach has been found to identify the GO terms that are significantly enriched (Tatusov et al., 2000).

#### 2.6.3 KOG annotation and functional classification of DEGs

The KOG (eukaryotic orthologous group) is a taxonomy of euryarchaeal proteins that are classified into groups based on sequence similarity. Each group is assigned a KOG number that represents homologous proteins (Wang et al., 2019). In this study, euryarchaeal homologous proteins were clustered, and their genes were annotated. To acquire relevant annotation information, sequences were compared to the KOG database using a basic local alignment search tool (BLAST) program to acquire relevant annotation information.

### 2.7 Weighted gene co-expression network analysis

#### 2.7.1 Construction and clustering of gene networks

The WGCNA was applied to the DEGs for the purpose of identifying the modules and genes associated with anthocyanin synthesis. Initially, the expression matrix of the DEGs was loaded and examined for any missing values, with details provided by the good sample genes. Subsequently, the samples were clustered, and a soft threshold power β was selected to compute the weight parameters of the neighbor-joining matrix. This step facilitated the construction of the network, the identification of modules, and the plotting of a heatmap illustrating module-trait correlations (Xie et al., 2013).

#### 2.7.2 Identify the hub genes

The identification of modules with the strongest correlation to anthocyanin content was achieved through the implementation of the Gene-Module Membership (MM) and Gene Significance (GS) metrics. The MM value of each gene in the module with a strong correlation to anthocyanin content was first obtained by calculating the average of the correlation between the gene and other genes in the module. Subsequently, the GS value of each gene with anthocyanin content was calculated by the correlation coefficient. Following this, a comprehensive evaluation of the MM and GS values was conducted to identify the genes with high MM and GS values. The Cytoscape recognizable files were exported (Chin et al., 2014). Finally, Cytoscape software was employed to construct the expression network map, and the CytoHubba plug-in was utilized to identify hub genes (Moebes et al., 2022).

### 2.8 Verification of DEGs by RT-qPCR

#### 2.8.1 Synthesis of reverse transcriptional cDNA of passion fruit

A total of ten hub genes were identified in the turquoise and blue modules by WGCNA. To assess the accuracy of the WGCNA strategy, these genes were then validated using quantitative PCR (Xiaohan et al., 2023). The RNA extraction process was initiated with the use of a total RNA extraction kit (Tiangen Biotech (Beijing) Co., Ltd., China). Subsequently, the synthesis of cDNA was facilitated through reverse transcription, employing the WX2050 SuperScript cDNA Synthesis Kit (Beijing Huayueyang Biological Technology Co., Ltd., China) (Tian et al., 2018).

#### 2.8.2 RT-qPCR

The cDNA obtained from the reverse transcription reaction was then subjected to real-time fluorescent quantitative PCR (qPCR) using the Hieff qPCR SYBR Kit (Yisheng Biotechnology Co., Ltd., Shanghai, China) (Zhao et al., 2021). Primers for selected genes were designed using Primer Premier 5.0. Three biological replicates were performed for all samples. Subsequently, the relative expression levels of the genes of interest were calculated using the 2-ΔΔCT method (Suarez et al., 2017).

## 3 Results

### 3.1 Transcriptome sequencing and alignment

To investigate the molecular basis of anthocyanin accumulation in passion fruit peel, RNA-seq analysis was performed on purple and yellow varieties at two developmental stages. Samples were collected from mature and immature fruits, with three biological replicates for each group, totaling 12 groups. RNA-Seq was performed on the Illumina-HiSeq platform, generating 559 million raw reads. After filtering, 535 million clean reads with an average length of 150 bp were obtained. The raw sequence data was evaluated using the Fastqc, and the summary reports were generated using the MultiQC. Based on these evaluations, the data was further filtered or trimmed to remove reads including sequences with substandard adapter sequences, excessive N bases, low-quality bases, and repetitive sequences caused by non-random fragmentation. The filtered clean reads was then aligned to the passion fruit reference genome using HISAT2 (**Table 1**). The alignment results indicated that the alignment rate for each group was higher than 73%, indicating high data quality, good mapping efficiency and no contamination occurred during sequencing. The transcriptome data obtained in this sequencing can be used for subsequent analysis.

**Table 1:** The comparison results of the transcriptome with the reference genome.

Sample name	Clean reads	Mapped reads	Read1 mapped	Read2 mapped
TPU1	43477306	34108836(78.45%)	16436263(37.80%)	16423425(37.77%)
TPU2	48173982	37917091(78.71%)	18273868(37.93%)	18255871(37.90%)
TPU3	40623528	32221645(79.32%)	15531646(38.23%)	15508634(38.18%)
TPR1	42473880	31755867(74.77%)	15255067(35.92%)	15245220(35.89%)
TPR2	43862056	33316327(75.96%)	16011624(36.50%)	15999297(36.48%)
TPR3	44558662	34236170(76.83%)	16507808(37.05%)	16449649(36.92%)
TYU1	47840074	36151766(75.57%)	17442442(36.46%)	17414667(36.40%)
TYU2	42384956	33261890(78.48%)	15938716(37.60%)	15914709(37.55%)
TYU3	48477292	38004310(78.40%)	18217319(37.58%)	18186284(37.52%)
TYR1	44621880	32579244(73.01%)	15685576(35.15%)	15673975(35.13%)
TYR2	42243164	31615619(74.84%)	15269365(36.15%)	15245369(36.09%)
TYR3	46597392	34180054(73.35%)	16499204(35.41%)	16468314(35.34%)

### 3.2 Screening of differentially expressed genes

**Figure 1:**
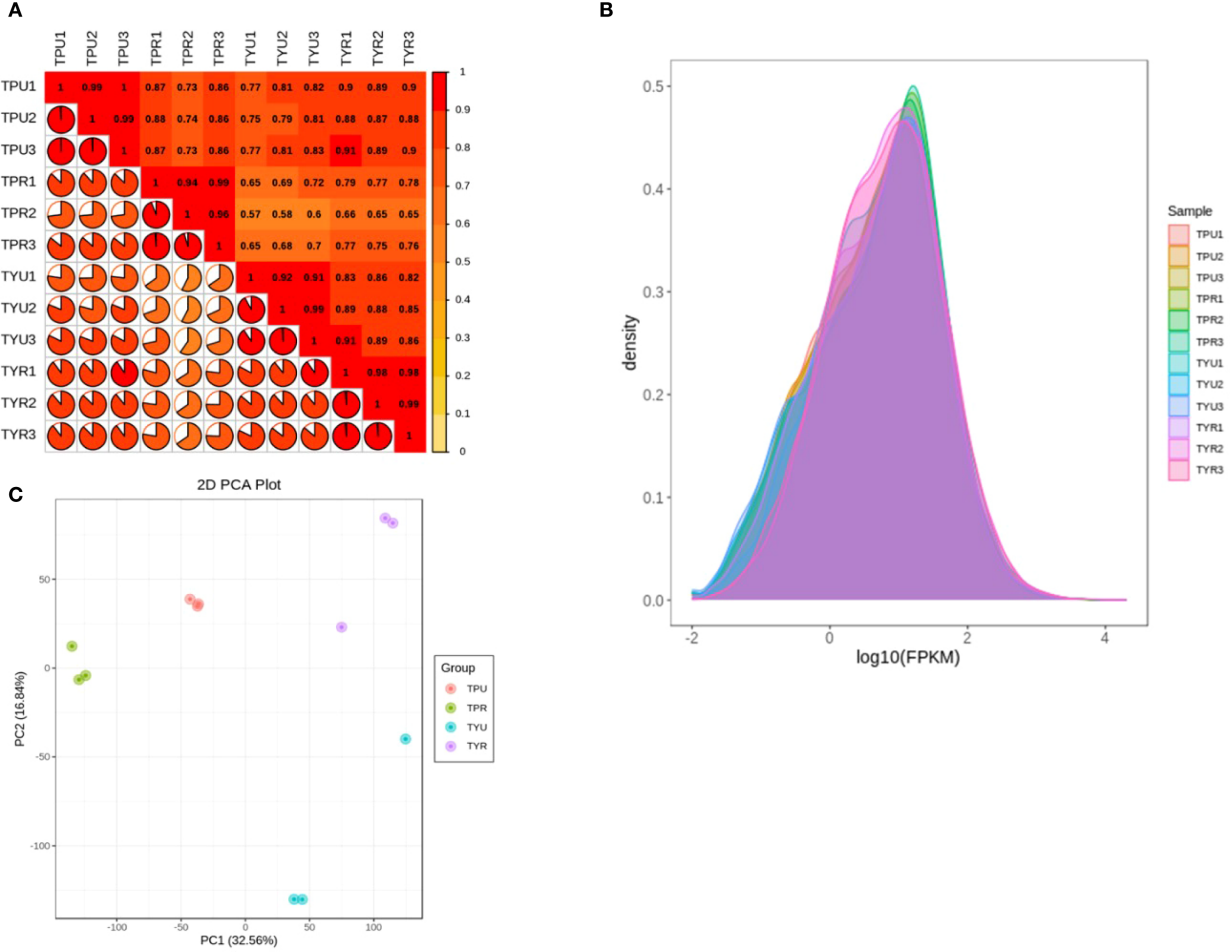
**(A)** Sample correlation analysis. **(B)** Comparison of sample gene FPKM density. **(C)** Principal component analysis of samples.

The correlation analysis of biological replicate samples requires that the R^2^ value between replicates should be at least 0.8 or higher. Statistical analysis (seen in **Figure 1A**) showed that, the R^2^ value among biological replicates in each group was greater than 0.9, which met the requirements of quality criteria and confirmed the reliability of the data for subsequent analysis. The FPKM density distribution of the 12 groups was compared (**Figure 1B**), and the results showed that there was a large overlap among samples, indicating that the overall gene expression levels of the samples were similar. The non-overlapping parts might be attributed to variations in the different varieties and maturity stages of the passion fruit, which caused changes in gene expression levels.

In the principal component analysis (**Figure 1C**), the scattered points corresponding to the four sample groups displayed a trend of mutual aggregation within each groups, suggesting good internal repeatability and high similarity among the sample data; In contrast, the scattered points between the groups exhibited a pattern of mutual dispersion, indicating distinct discrimination among the groups, thus suitable for downstream differential expression and functional analyses.

The R package DESeq2 was employed to analyze the differential expression between samples, resulting in the identification of differentially expressed gene sets across various group. After the differential analysis, the Benjamini-Hochberg method was applied to adjust the P-values for multiple hypothesis testing. By setting the criteria of |log_2_Fold Change| ≥ 1 and FDR < 0.05 as significance threshhold, the differentially expressed genes (DEGs) were screened, and the differences among genes between different combinations were also compared.

**Figure 2:**
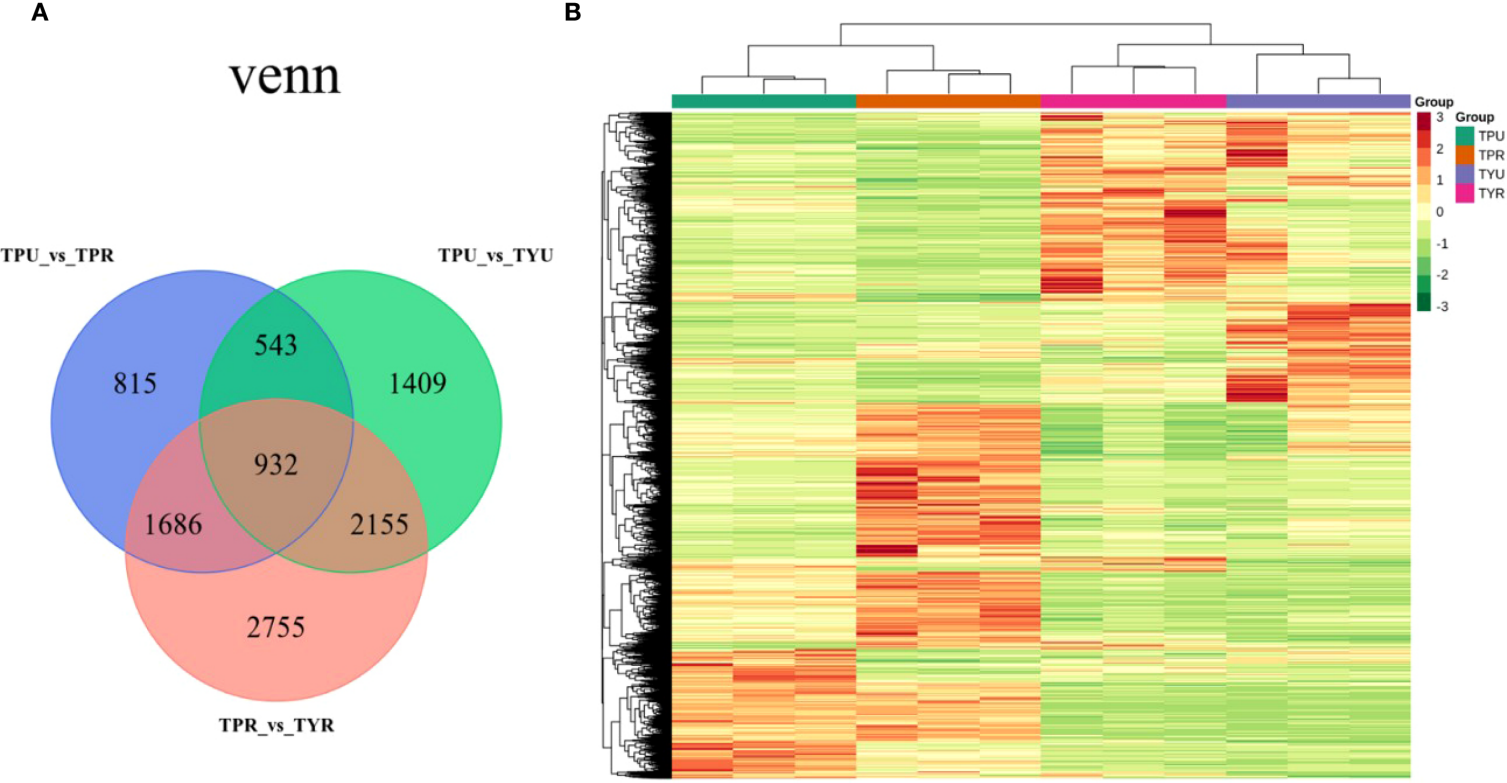
**(A)** Venn diagram. **(B)** clustering heatmap.

The Venn diagram (**Figure 2A**) illustrates the overlap of DEGs among different comparison groups, providing critical insights into the transcriptional regulation network underlying passion fruit peel coloration. Comparative analysis between mature purple-skin and yellow-skin passion fruit (TPR_vs_TYR) identified 7,528 DEGs, with 3,504 upregulated and 4,024 downregulated genes. This substantial change suggests variety-specific metabolic pathways are most active in mature fruit. Addtionally, fewer DEGs (5,039) were detected between immature purple-skin and yellow-skin fruit (TPU_vs_TYU), comprising 2,272 upregulated and 2,767 downregulated genes, indicating variety differences are already established early in development. This suggests that transcriptomic divergence increases along with fruit ripening, potentially corresponding to anthocyanin accumulation. Furthermore, a comparison between the immature stage of purple-skin passion fruit and the mature stage (TPU_vs_TPR) revealed 3,976 differentially expressed genes, including 1,894 up-regulated genes and 2,082 down-regulated genes, likely playing crucial roles in pigment accumulation during maturation.

Hierarchical clustering analysis was performed using normalized FPKM values of differentially expressed genes, and the resulting heatmap (**Figure 2B**) revealed distinctive expression patterns across samples. Notably, TPU and TPR samples clustered more closely together, as did TYU and TYR samples, indicating that genotype (purple-skin versus yellow-skin) exerts a stronger influence on global gene expression patterns than developmental stage. This genotype-dependent clustering suggests that the fundamental transcriptional programs governing fruit development are largely conserved within each variety across maturation stages. In contrast, there were marked differences in the color patterns between TPU and TYU, as well as between TPR and TYR. These differences in gene expression patterns between purple-skin and yellow-skin genotypes likely reflect divergent regulation of pigment biosynthesis pathways, particularly those involved in anthocyanin accumulation. The hierarchical clustering results provide a foundation for identifying the key regulatory modules that differentiate anthocyanin-accumulating and non-accumulating passion fruit genotypes.

### 3.3 KEGG annotation and enrichment analysis of DEGs

**Figure 3:**
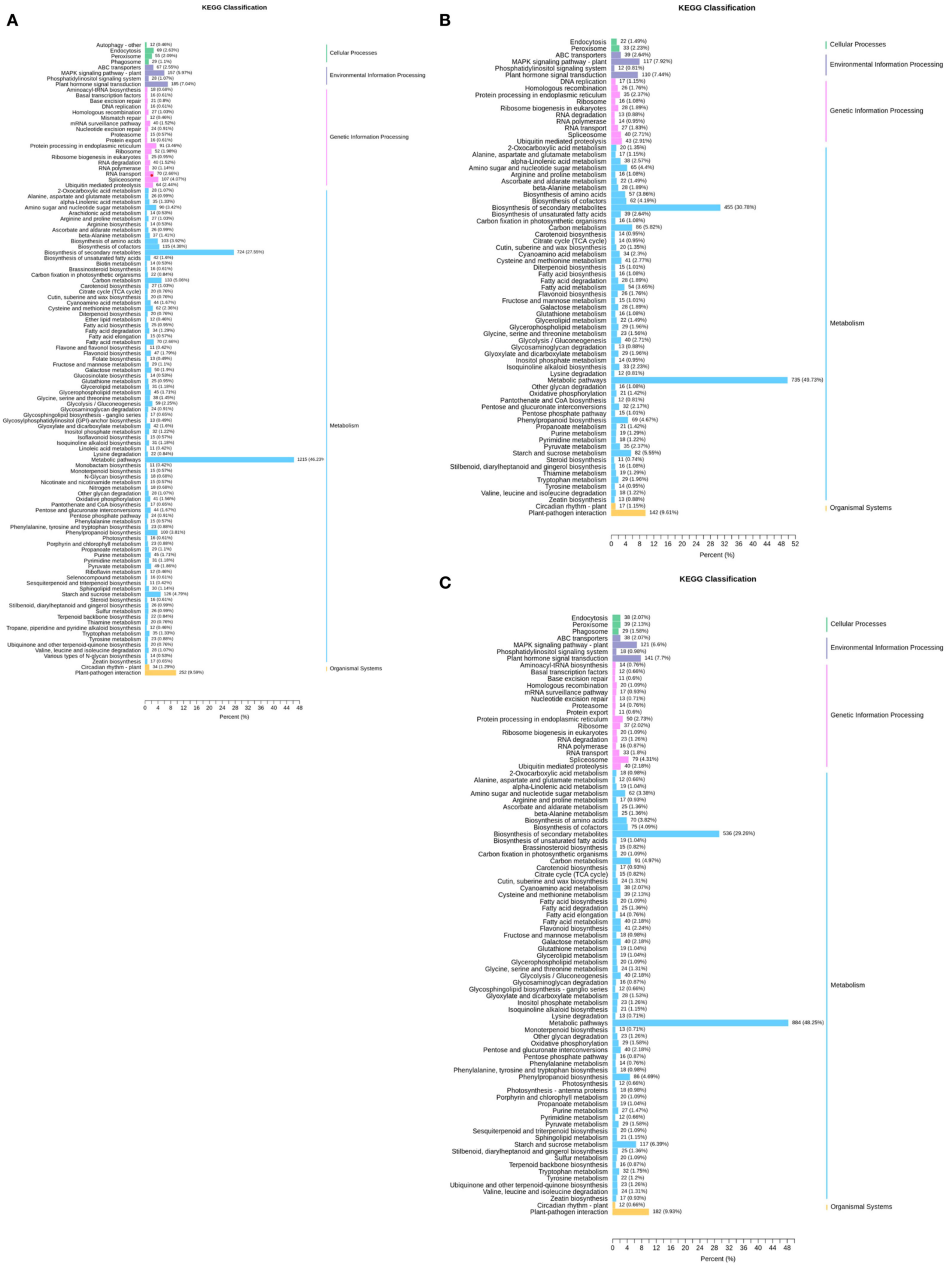
KEGG annotation of DEGs. **(A)** TPR_vs_TYR. **(B)** TPU_vs_TPR. **(C)** TPU_vs_TYU.

To elucidate the biological functions of DEGs, KEGG pathway enrichment analysis was implemented (**Figure 3**). In the comparison of TPR_vs_TYR (**Figure 3A**), 2628 of the 7528 DEGs were successfully annotated, accounting for 34.91% of the total DEGs, and they were involved in 113 metabolic pathways of KEGG. The largest number of annotated genes was found in the category of metabolism, with the metabolic pathway pathways and the biosynthesis of secondary metabolites having the most annotated DEGs, totaling 1215 and 724, respectively.

For the comparison of TPU_vs_TPR (**Figure 3B**), KEGG annotation was conducted on 3,976 DEGs, revealing that 1,478 of these genes were annotated, which accounted for 37.17% of the total DEGs, and they were involved in 72 KEGG metabolic pathways. Again, the majority of annotated genes were in the metabolism category, with 735 DEGs related to metabolic pathways and 455 to the biosynthesis of secondary metabolites.

Additionally, KEGG annotation was performed on 5,039 DEGs from the TPU_vs_TYU comparison (**Figure 3C**), where 1,832 of the DEGs were annotated, representing 36.36% of the total DEGs, and these genes were involved in 89 KEGG metabolic pathways. As with the previous comparisons, the largest number of annotated genes was in the metabolism category, with 884 DEGs associated with metabolic pathways and 536 with the biosynthesis of secondary metabolites.

**Figure 4:**
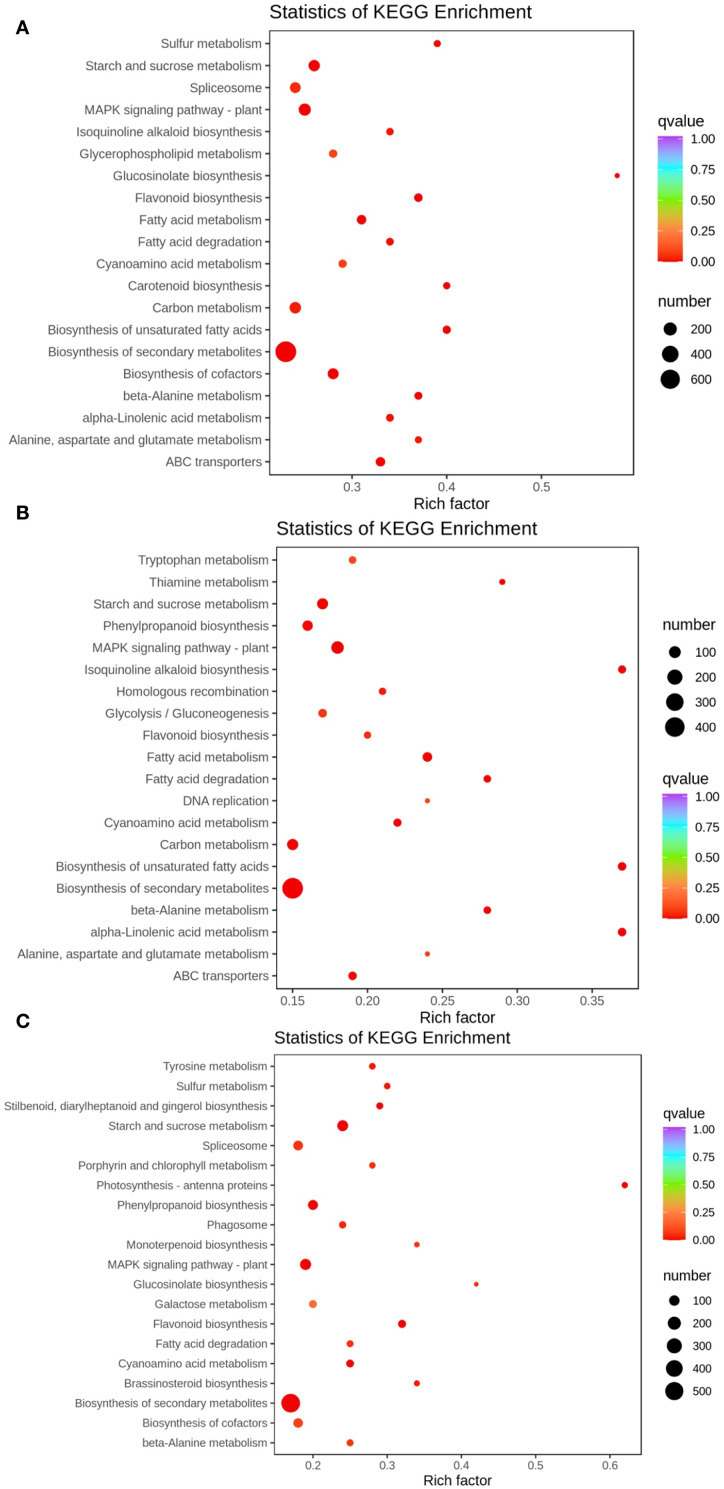
Enrichment map of DEGs in KEGG pathways. **(A)** TPR vs TYR, **(B)** TPU vs TPR, **(C)** TPU vs TYU.The color of the dots represents the confidence level of enrichment, and the size of the dots represents the number of enriched genes.

After the genes have been annotated, the number of DEGs in each pathway was calculated, and a KEGG enrichment scatter plot of DEGs was drawn (**Figure 4**). The KEGG enrichment scatter plot for the DEGs from the TPR_vs_TYR compariso (**Figure 4A**) display the 20 most significantly enriched pathway entries, with the DEGs annotated to the biosynthetic pathways of secondary metabolites being the most numerous, far exceeding other pathways. Therefore, the Rich factor of this pathway is the smallest and the q-value is the lowest, indicating significant enrichment.

Similarly, the KEGG enrichment scatter plot for the DEGs from the TPU_vs_TPR comparison (**Figure 4B**) also shows the 20 most significantly enriched pathway entries, where the DEGs associated with the biosynthetic pathways of secondary metabolites are again the most numerous. This pathway exhibits the smallest Rich factor and the lowest q-value, confirming its significant enrichment.

Furthermore, the KEGG enrichment scatter plot for the DEGs from the TPU_vs_TYU comparison (**Figure 4C**) presents the 20 most significantly enriched pathway entries, with the DEGs annotated to the biosynthetic pathways of secondary metabolites remaining the most numerous. As with the previous comparisons, this pathway has the smallest Rich factor and the lowest q-value, indicating significant enrichment.

### 3.4 GO and KOG annotation of DEGs

**Figure 5:**
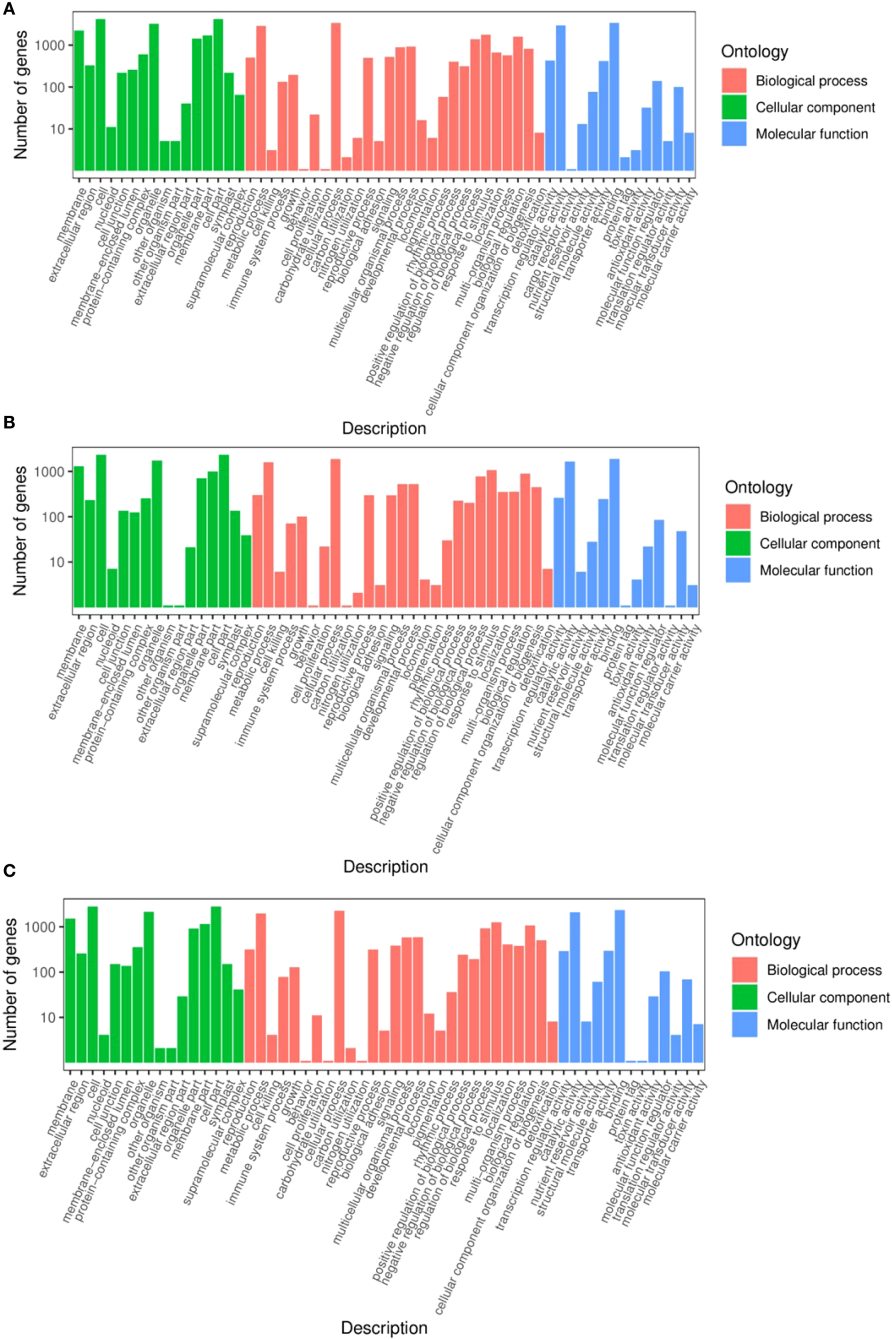
GO annotation of DEGs. **(A)** TPR_vs_TYR, **(B)**TPU_vs_TPR, **(C)** TPU_vs_TYU.

In the GO term enrichment analysis, 7528 DEGs from the TPR_vs_TYR comparison were categorized into 58 subclasses across the three broad categories (**Figure 5A**). Within the BPs category, the categories were “Cellular Processes” (45.1%), “Metabolic Processes” (38.2%), and “Response to Stimulus” (23%). The subclasses annotated under the CCs category included “Cells” (55.6%), “Organelles” (43%), and “Membranes” (29.4%). In the MFs category, the annotations were predominantly “Binding” (45%) and “Catalytic Activity” (39.1%).

In the TPU_vs_TPR comparison, 3976 DEGs were categorized into 56 subclasses across the three broad categories (**Figure 5B**). For the BPs category, the distribution was as follows: “Cellular Processes” (47.2%), “Metabolic Processes” (40.1%), and “Response to Stimulus” (26.8%). In the CCs category, the DEGs were annotated to “Cells” (58.6%), “Organelles” (43.4%), and “Membranes” (32.8%). For the MFs category, the predominant subclasses were “Binding” (47.5%) and “Catalytic Activity” (41.5%).

In the TPU_vs_TYU comparison, 5039 DEGs were classified into 57 subclasses within the three broad categories (**Figure 5C**). Within the category of BPs, the most prevalent subclasses were “Cellular Processes” (45.1%), “Metabolic Processes” (38.3%), and “Response to Stimulus” (25.1%). For the CCs category, the annotations were “Cells” (55.6%), “Organelles” (42.6%), and “Membranes” (30.1%). Finally, the MFs category was included “Binding” (45.6%) and “Catalytic Activity” (41.5%).

**Figure 6:**
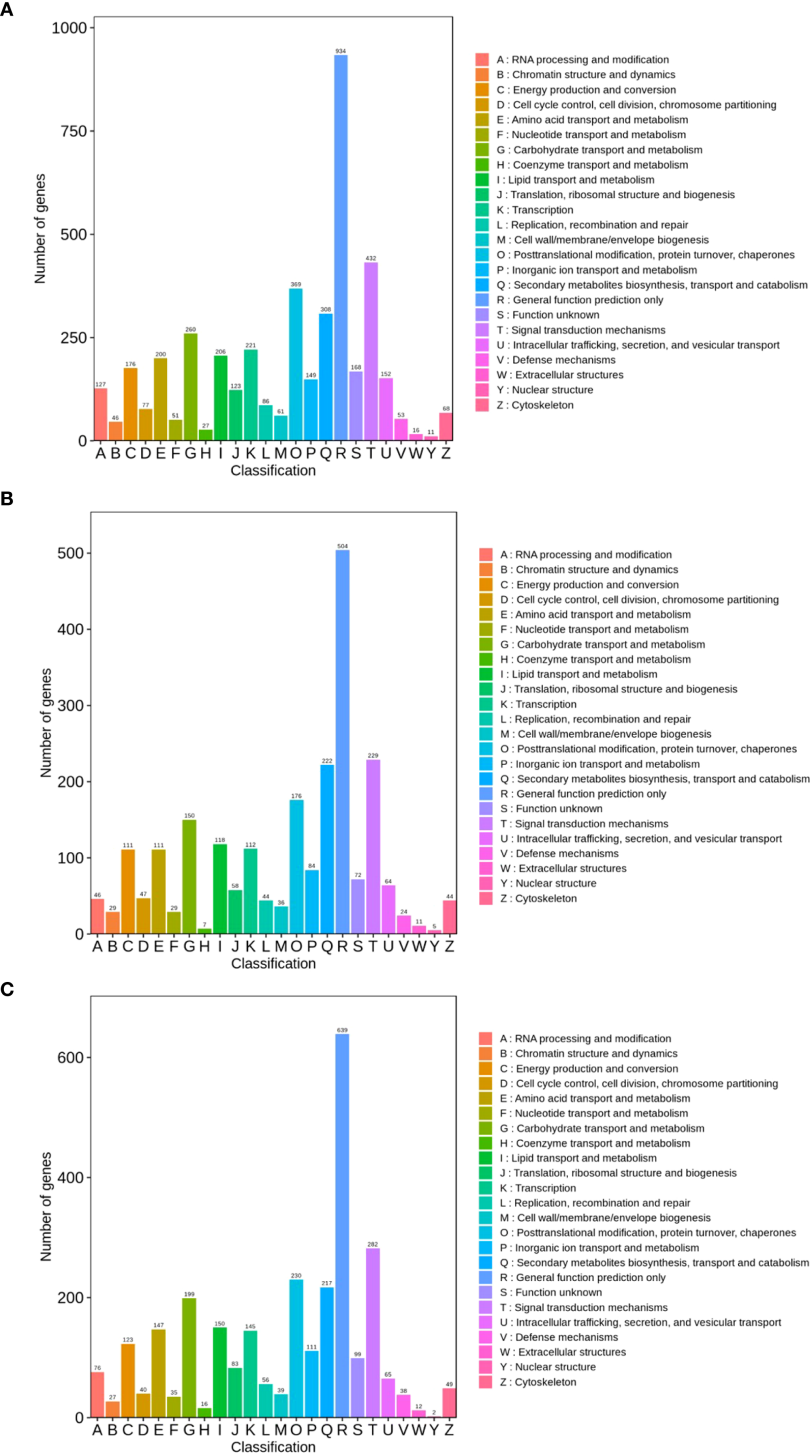
KOG annotation of DEGs. **(A)** TPR_vs_TYR, **(B)** TPU_vs_TPR, **(C)** TPU_vs_TYU.

In the TPR_vs_TYR comparison, 4321 DEGs were annotated to KOGs. As illustrated in **Figure 6A**, the five most prevalent KOG classifications are “Function Prediction” (934, 12.41%), “Transduction Machinery” (432, 5.74%), “Post-Translational Modifications, Protein Turnover, Molecular” (396, 4.90%), “Biosynthesis of Secondary Metabolites, Transport and Metabolism” (308, 4.09%), and “Carbohydrate Transport and Metabolism” (260, 3.45%).

In the TPU_vs_TPR comparison, 2333 DEGs were annotated to the KOG database. As illustrated in **Figure 6B**, the top five KOG classifications are as follows: “Function Prediction” (504, 12.68%), “Transduction Mechanism” (229, 5.76%), “Secondary Metabolite Biosynthesis, Transport and Metabolism” (222, 5.58%), “Post-Translational Modifications, Protein Turnover, Molecular” (176, 4.43%), and “Carbohydrate Transport and Metabolism” (150, 3.77%).

In the TPU_vs_TYU comparison, 2880 DEGs were annotated to KOGs. As illustrated in **Figure 6C**, the five most prevalent KOG classifications are: “Function Prediction” (639, 12.68%), “Transduction Mechanism” (282, 5.60%), “Post-Translational Modifications, Protein Turnover, Molecular” (230, 4.56%), “Biosynthesis of Secondary Metabolites, Transport and Metabolism” (217, 4.31%), and “Carbohydrate Transport and Metabolism” (199, 3.95%).

### 3.5 Weighted gene co-expression network analysis

**Figure 7:**
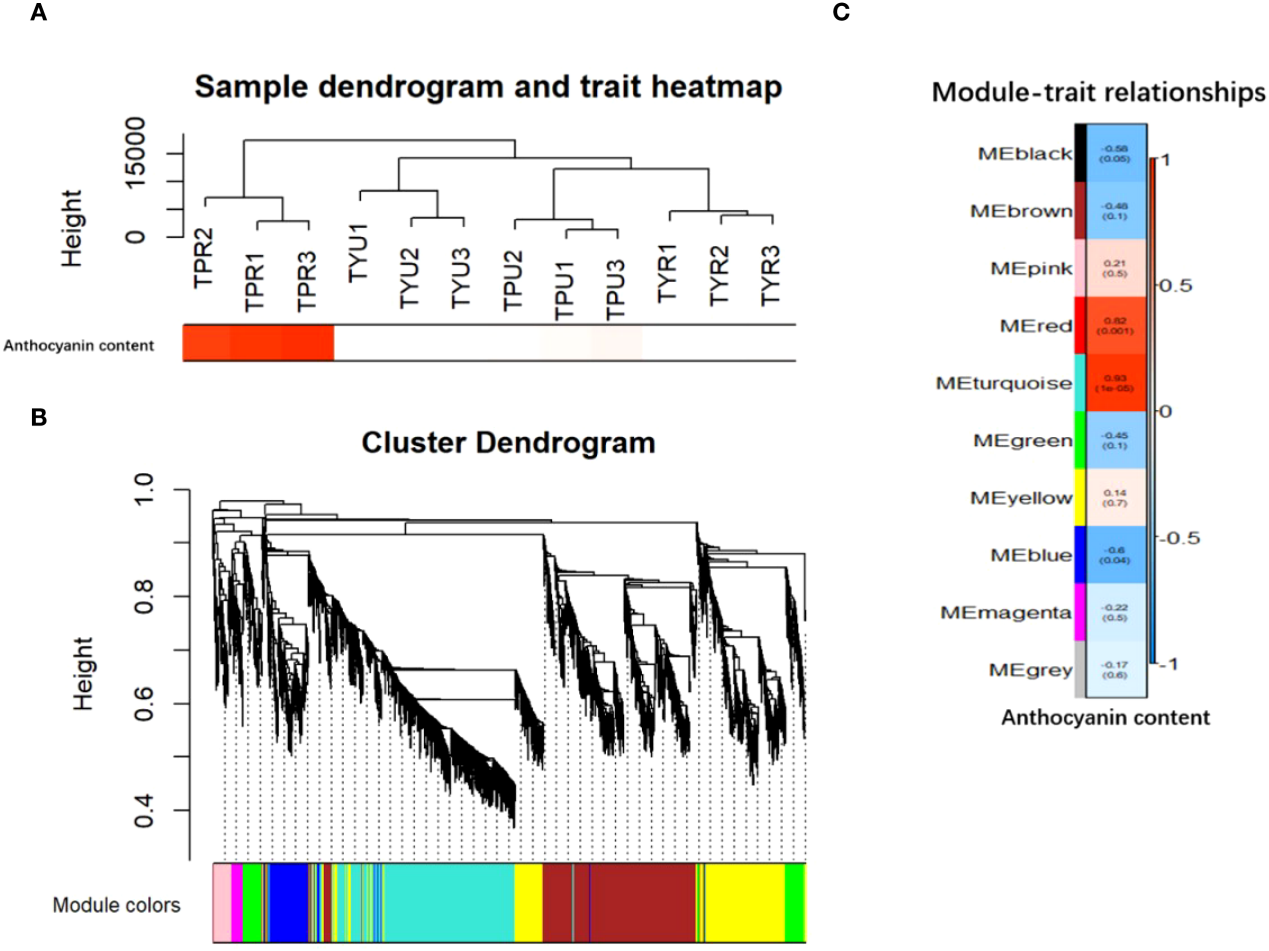
**(A)** Heatmap of sample and trait clustering. **(B)** Hierarchical clustering tree diagram and module identification. **(C)** Correlation heat map between sample module and trait,The color block on the far left represents the module, and the color bar on the far right represents the range of correlation. In the heatmap in the middle section, the darker the color, the higher the correlation. Red indicates positive correlation, and blue indicates negative correlation. The number in each cell represents the correlation and significance.

This study employed weighted gene co-expression network analysis (WGCNA) to cluster the DEGs in the peel samples of purple and yellow passion fruit at varying maturities. The genes were organized into multiple modules, which were found to be associated with anthocyanin content. As demonstrated in **Figure 7A**, the clustering of DEGs was executed using anthocyanin content as an external trait. A heat map was generated to illustrate the sample clusters for this trait, revealing that TPR had the highest correlation. The construction of the co-expression network was facilitated by employing an optimal soft threshold, a process that enabled the aggregation of genes into discrete modules. The construction of the gene clustering tree was performed to cluster the distance between these modules, which also illustrates their relationships.

As illustrated in **Figure 7B**, the upper portion of the figure presents the hierarchical clustering tree of the genes, while the lower portion illustrates the gene modules. The genes that exhibited strong relatedness were observed to cluster together and be assigned to the same module. The heat map illustrates the modules as color blocks on the left and the corresponding ranges as a color bar on the right. In the central section, darker colors indicate stronger correlations, with red signifying positive correlations and the blue denoting negative correlations. The numerical values within each cell denote the degree of correlation and its statistical significance. The results indicated a positive correlation between the turquoise module and anthocyanin content, while a negative correlation was observed between the blue module and the same variable. Subsequent analyses were conducted on the anthocyanin content related to the turquoise module. To identify the genes with a high membership in the turquoise module, the “Gene Significance” (GS) and “Module Membership” (MM) metrics were employed.

As illustrated in **Figure 7C**, the gene that demonstrates a high degree of correlation with a specific trait exhibits a robust correlation between “Gene Significance” (GS) and “Module Membership” (MM) within its designated module. This finding suggests that these genes play crucial roles within the key modules.

**Figure 8:**
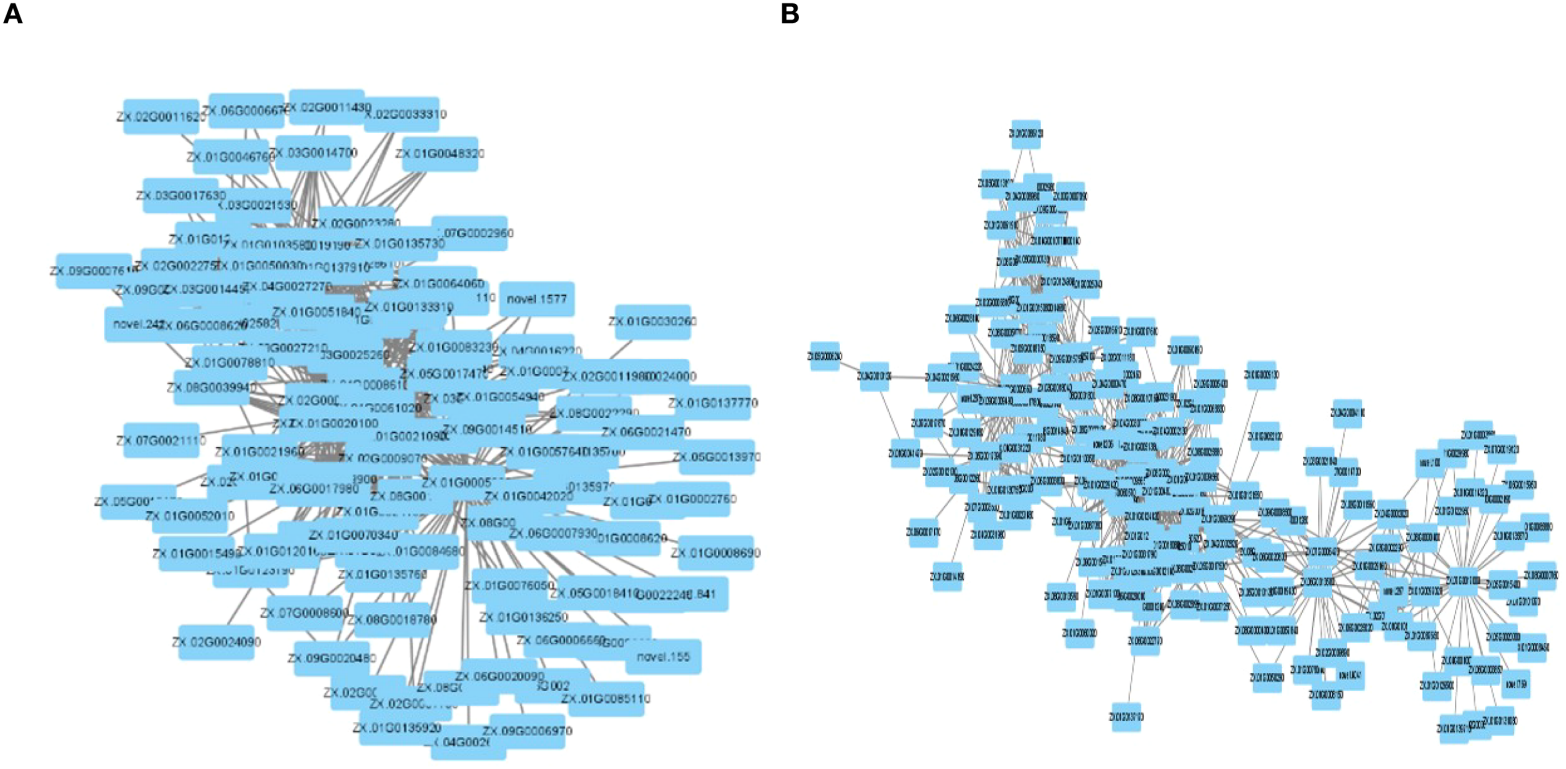
**(A)** turquoise module co-expresses network diagram. **(B)** blue module co-expresses network diagram.

The construction of the gene co-expression network for the two related modules was facilitated by the Cytoscape software. The turquoise module identified 5 hub genes (**Figure 8A**), and the blue module similarly identified 5 hub genes (**Figure 8B**) using the MCC algorithm.

### 3.6 RT-qPCR verification of DEGs

**Figure 9:**
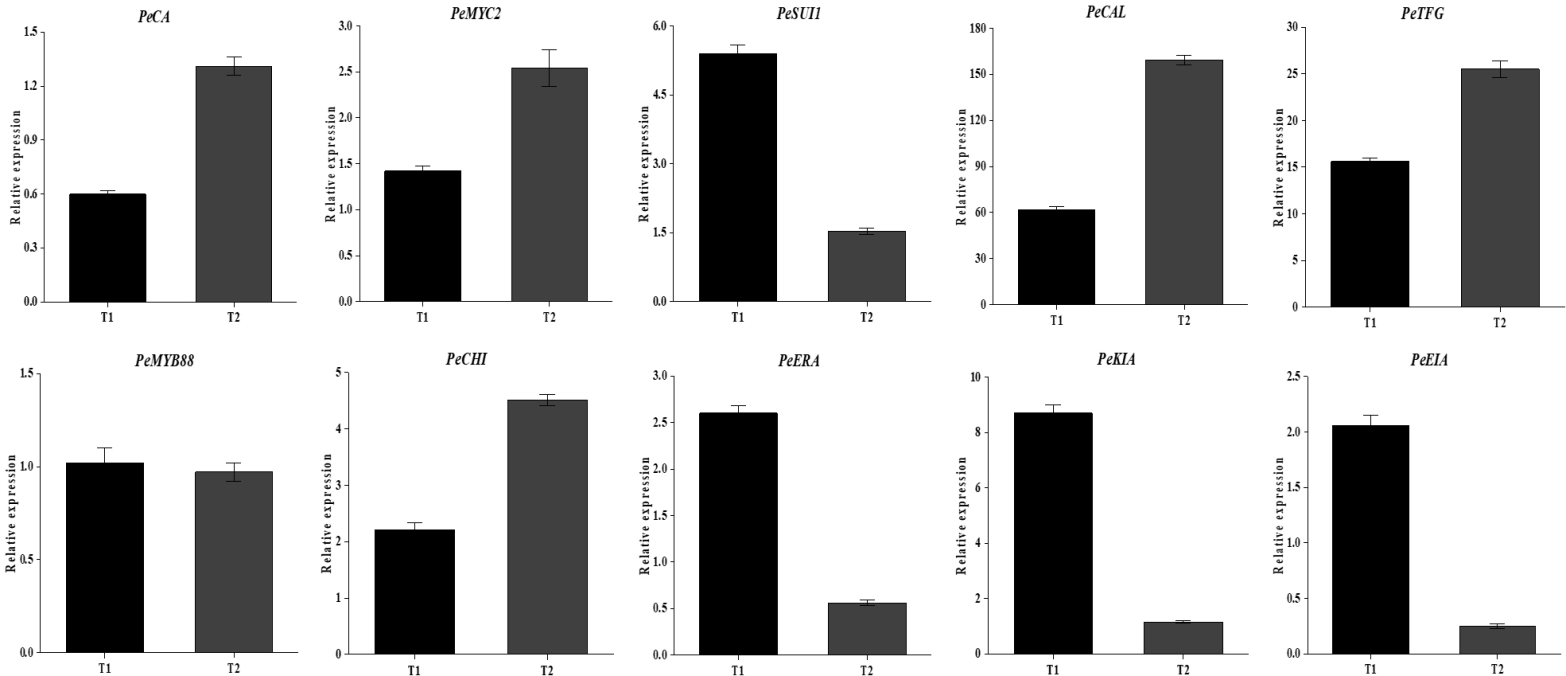
Relative expression levels of key genes. T1 represents the immature stage, while T2 represents the mature stage.

**Figure 10:**
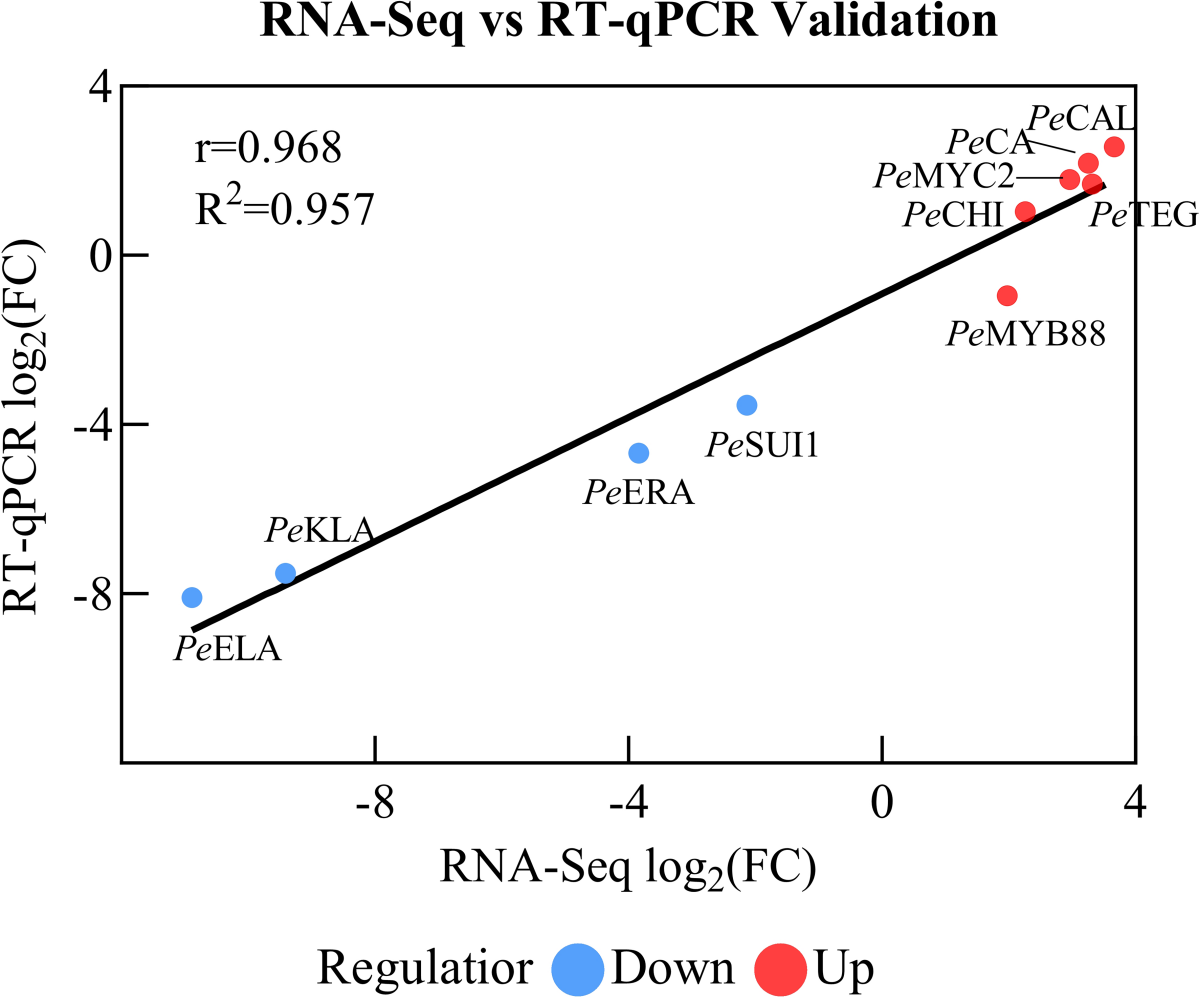
Validation of the correlation between RNA-Seq and RT-qPCR results.

In order to verify the reliability of the transcriptional sequencing data, 10 genes related to anthocyanin content were selected for a real-time fluorescence quantitative PCR verification (**Table 2**). The β-actin gene was utilized as an internal reference for the test samples by SYBR@Green I chimeric fluorescence method to detect the relative expression levels of each factor at the two maturity levels (**Figure 9**). The relative expression levels of RT-qPCR were calculated using the 2-ΔΔCT method, while the RNA-seq data were represented by the log2-transformed FPKM values. We conducted a correlation analysis on these two datasets. The results are shown in **Figure 10**, with a Pearson correlation coefficient (r = 0.968 > 0.9, *p* < 0.001), indicating that the two methods have extremely strong consistency in detecting gene expression changes. This high consistency confirms the reliability and accuracy of our transcriptome data.

**Table 2:** Comparison of transcriptome data and RT-qPCR results.

Gene ID	Name	Definition	RNA-seq	RT-qPCR
ZX.01G0005290	*PeCA*	4-coumarate-CoAligaseactivity	3.26	2.18
ZX.09G0013530	*PeMYC2*	transcription factor MYC2	2.96	1.79
ZX.01G0010830	*PeSUI1*	translation initiation factor SUI1	-2.13	-3.54
ZX.01G0005530	*PeCAL*	catalytic activity, acting on a protein	3.67	2.57
ZX.01G0038770	*PeTFG*	transferase activity, transferring glycosyl groups	3.32	1.68
ZX.08G0032360	*PeMYB88*	transcription factor MYB88	1.98	-0.95
ZX.01G0100390	*PeCHI*	Chalcone isomerase	2.26	1.04
ZX.01G0037350	*PeERA*		-3.84	-4.67
ZX.01G0010090	*PeKIA*	kinase inhibitor activity	-9.41	-7.52
ZX.08G0027120	*PeEIA*	enzyme inhibitor activity	-10.88	-8.09

The results demonstrated the up-regulation of *PeCA* (4-coumarate-CoA ligase activity), *PeMYC2* (transcription factor MYC2), and *PeCHI* (chalcone isomerase) gene expression and the down-regulation of *PeKIA* (kinase inhibitor activity) and *PeEIA* (enzyme inhibitor activity) expressions. Consequently, the expression of these genes was found to be instrumental in the promotion of anthocyanin accumulation and synthesis.

## 4 Discussion

Anthocyanin,a water-soluble pigment commonly present in dark-colored plants, plays a key role in determining the color of plant fruits or flowers. Numerous studies have demonstrated that variations in anthocyanin content directly influence the visual presentation of plant colors.

The accumulation of anthocyanins is a hallmark of fruit maturation in purple passion fruit, yet the underlying molecular architecture remains poorly understood. By integrating RNA-seq and WGCNA, this study dissects the complex regulatory network governing this process, moving beyond individual gene analysis to reveal a multi-layered control system. Our findings pinpoint key transcriptional regulators, crucial enzymatic steps, and a potentially novel post-translational control mechanism, collectively orchestrating anthocyanin biosynthesis in *Passiflora edulis* peel.

The anthocyanin biosynthetic pathway, branching from the phenylpropanoid pathway, has been well-characterized across various plant species. This pathway necessitates coordinated expression of structural genes encoding biosynthetic enzymes, which is primarily controlled by a transcriptional regulatory complex comprising R2R3-MYB, basic helix-loop-helix (bHLH), and WD40 repeat proteins (MBW complex) (DiLeo et al., 2011). Our WGCNA analysis effectively captured this regulatory framework, Identification of key genes involved in anthocyanin biosynthesis and their associated transcription factors:

(i) positive regulatory factor

Among the identified hub genes, *PeCA* (encoding 4-coumarate:CoA ligase, 4CL) emerged as a critical regulatory node. The 4CL enzyme catalyzes the conversion of p-coumaric acid into 4-coumaroyl-CoA, providing essential precursors for downstream flavonoid biosynthesis. Our findings indicated that *PeCA* expression was positively correlated with anthocyanin content, consistent with previous findings in blueberries. In blueberries, enhanced 4CL activity promoted both lignin and anthocyanin accumulation through upregulation of the phenylpropanoid pathway (Xie et al., 2018). Recent studies have further confirmed the crucial role of 4CL in flavonoid biosynthesis, demonstrating that its activity directly influences anthocyanin accumulation (Keller-Przybylkowicz et al., 2024; Golovatskaya et al., 2024).

At the apex of the regulatory hierarchy are transcription factors (TFs) that orchestrate the expression of the entire pathway. Our WGCNA results strongly support the operation of this complex in passion fruit. We identified *PeMYC2*, encoding a bHLH transcription factor, as a key regulator within the anthocyanin-associated modules. MYC2 transcription factors have been extensively studied in jasmonate signaling pathways, where they govern the production of secondary metabolites, including anthocyanins. Significantly, the disruption of MYC2 function has been shown to impair anthocyanin regulation in apple fruit (Sun et al., 2019), highlighting its conserved role across species. The high connectivity of *PeMYC2* in our co-expression network suggests it may function as a central integrator, linking stress signals to anthocyanin biosynthesis in passion fruit.

Similarly, *PeMYB88* was identified as another hub transcription factor in our analysis. R2R3-MYB transcription factors is known as one of the largest regulatory families in plants and have been well-documented as regulators of anthocyanin biosynthesis (Ma et al., 2022). Recent studies in chili pepper demonstrated that transient overexpression of *CaMYB5* resulted in significant anthocyanin accumulation, accompanied by upregulation of key biosynthetic genes including *Ca4CL* and *CaCHI* (Zhou et al., 2025). *PeMYB88* is highly homologous to *CaMYB5* and has a close genetic relationship. This indicates that *PeMYB88* regulates the synthesis of anthocyanins by modulating the expression of anthocyanin biosynthesis-related structural genes such as *Pe4CL* and *PeCHI.*

(ii) negative regulatory factors

Intriguingly, our analysis also revealed a downregulation of genes encoding enzyme inhibitors (*PeKIA* and *PeEIA*) in samples with high anthocyanin content. In the biosynthetic pathway of anthocyanins, the expressions of enzymes such as PAL, CHI and F3H have a significant positive correlation with the synthesis of anthocyanins. This suggests a novel regulatory mechanism in which decreased inhibitor activity may enhance the enzymatic flux through the anthocyanin biosynthetic pathway, For instance, sugars phosphorylated by hexokinase can induce the expression of F3H, thereby increasing the accumulation of anthocyanins. Meanwhile, the specific inhibitors of hexokinase, such as glucosamine and mannose heptose, can block this induction process (Zheng et al., 2009). Although, post-translational regulation through enzyme inhibitors has been less studied in anthocyanin biosynthesis compared to transcriptional control, our findings highlight its potential importance and warrant further investigation.

(iii) enzymatic reactions

At the enzymatic level, significant upregulation of *PeCHI* (chalcone isomerase), which catalyzes the stereospecific conversion of chalcones to flavanones—a critical early step in flavonoid biosynthesis, was observed. The positive correlation between *PeCHI* expression and anthocyanin content is consistent with previous reports that have been demonstrated the rate-limiting nature of this enzymatic step (Guo et al., 2015). Recent functional characterization of chalcone isomerases from other species has confirmed their essential role in anthocyanin biosynthesis (Nakanishi et al., 2024), thus supporting our findings in passion fruit.

The integrated transcriptome and WGCNA analyses provide a comprehensive perspective on the anthocyanin regulatory network in passion fruit peel. The identified hub genes cover multiple regulatory levels, ranging from precursor supply (*PeCA*) through transcriptional control (*PeMYC2*, *PeMYB88*) to enzymatic catalysis (*PeCHI*) and post-translational regulation *(PeKIA*, *PeEIA*). This multi-layered regulatory framework likely enables precise control of anthocyanin accumulation in response to developmental and environmental signals.

In summary, this study enhances our understanding of anthocyanin biosynthesis regulation in passion fruit peel through integrated transcriptomic and network analyses. The identified regulatory genes and their co-expression patterns provide valuable targets for genetic improvement of fruit color and nutritional quality in passion fruit breeding programs.

## 5 Conclusion

In this paper, Firstly, we compared the differentially expressed genes involved in anthocyanin synthesis among various degrees of ripeness of passion fruits. We annotated these differentially expressed genes, analyzed their functions and metabolic pathways, and constructed a co-expression network of related modules and genes. Based on this, we identified potential hub genes related to anthocyanin synthesis. Finally, we verified the results of the transcriptome analysis through RT-qPCR, further discovering key genes related to anthocyanin synthesis, and providing reference bases for the subsequent research on the regulatory mechanism and functional identification of related genes. This study provides a reference basis for subsequent research on the regulatory mechanism of anthocyanin biosynthesis in passion fruit pericarp and the functional identification of related genes.

## Data Availability

The transcriptome sequence of passion fruit pericarp has been submitted to SRA (http://www.ncbi.nlm.nih.gov/sra/) under the accession number PRJNA1165243.
